# Do Moral Views Change during a Crisis? An Experiment on Health Care Priority Setting

**DOI:** 10.1177/0272989X251391177

**Published:** 2025-12-11

**Authors:** Liam Strand, Erik Gustavsson, Gustav Tinghög

**Affiliations:** Swedish National Centre for Priorities in Health, Department of Health, Medicine, and Caring Sciences, Linköping University, Sweden; Swedish National Centre for Priorities in Health, Department of Health, Medicine, and Caring Sciences, Linköping University, Sweden; Division of Philosophy and Applied Ethics, Department of Culture and Society, Linköping University, Sweden; Swedish National Centre for Priorities in Health, Department of Health, Medicine, and Caring Sciences, Linköping University, Sweden; Division of Economics, Department of Management and Engineering, Linköping University, Sweden

**Keywords:** crisis exceptionalism, scarcity theory, moral consistency, abstractness

## Abstract

**Background:**

Resource scarcity during large-scale crises, such as pandemics, can increase the emphasis on efficiency in medical decision making. However, it remains unclear whether such shifts are primarily driven by the direct experience of scarcity or by the way in which ethical principles for health care priority setting are expressed in the context of a crisis. This study investigates whether a national crisis affects public support for health care priority-setting principles and whether abstract versus concrete formulations of these principles shape that support.

**Design:**

We conducted a preregistered online experiment (*N* = 1,404) to examine public attitudes toward three ethical principles formalized in the Swedish ethical platform—human dignity, needs-solidarity, and cost-effectiveness—in both crisis and noncrisis contexts. We also manipulated how the principles were presented, using either abstract or concrete formulations.

**Results:**

In the crisis condition, support for the human dignity and cost-effectiveness principles decreased, while support for the needs-solidarity principle increased. However, these effects were small, and the overall ranking of the principles remained stable. Notably, the level of abstractness had a stronger impact than the crisis context did: support for needs solidarity was higher when described abstractly, whereas support for cost-effectiveness increased when it was presented in a more concrete, action-oriented way. Support for the human dignity principle was unaffected by the abstractness manipulation.

**Conclusion:**

The findings suggest that people’s moral views are relatively stable in the face of crisis. Rather than the crisis context itself, the way ethical principles are formulated—abstractly or concretely—may be a more powerful driver of shifts in public support for different moral values in health care priority setting.

**Highlights:**

- Public support for ethical principles remained largely stable during a simulated national crisis.
- The level of abstraction in how principles were presented strongly influenced support.
- Support for needs-solidarity increased in a crisis, while cost-effectiveness support declined.
- The way ethical principles were formulated had a greater impact than the presence of a crisis.

## Introduction

The world regularly faces large-scale crises, including natural catastrophes, pandemics, wars, and other human emergencies. These situations place pressure on critical health priority decisions. A widely accepted tenet of normative ethics is that moral standards should remain consistent across similar situations, unless justified by relevant differences.^1^ Yet, empirical research shows that people often struggle to apply ethical principles consistently.^2–5^ In the literature, triage has frequently been described as a tension between utilitarian and egalitarian values.^6–12^ In times of crisis, the former (efficiency-oriented values) often take precedence over the latter (equality-oriented values).^13,14^ This tension could also be observed in policy making during the COVID-19 pandemic,^15–18^ although the relation between these guidelines and the underlying theories about distributive justice may be more complex.^19^ The tendency to abandon otherwise reasonable principles during crises—a phenomenon we refer to as *crisis exceptionalism*—raises important ethical and psychological questions. One defining feature of crises is heightened scarcity, whether of time, money, or medical resources, which intensifies the need for prioritization.^15,16,20^ Scarcity may thus contribute to the perceived justification for crisis exceptionalism. However, previous research suggests that the way ethical principles are formulated also plays a crucial role: concretely phrased principles tend to increase public support for efficiency over equality,^5,21–23^ which may help explain why crisis guidelines often emphasized efficiency. In this study, we conduct a behavioral experiment to examine whether public support for core ethical principles for health care priority-setting remain consistent in the face of a national crisis and how the abstractness or concreteness of those principles influences that support.

Experiences from the COVID-19 pandemic suggest that policymakers often prioritized efficiency over equality as resource scarcity intensified.^15,17^ Behavioral research supports this, showing that scarcity can significantly influence decision making and cognitive functioning—a core idea in scarcity theory.^24^ On the one hand, scarcity can heighten the awareness of trade-offs, and thereby facilitate more goal-oriented decisions.^24–26^ On the other hand, scarcity can also impair cognitive function and induce a mindset in which focus is shifted away from defined long-term goals toward more attention-grabbing matters, leading to long-term adverse outcomes^24,27–30^ and reduced empathic concerns.^31^ In health care settings, this can manifest as a focus on maximizing the number of lives saved or minimizing total health loss—objectives often aligned with efficiency principles such as cost-effectiveness. At the same time, scarcity may reduce support for equality-oriented principles that emphasize need-based claims and priority to the worse off, as these require consideration of more abstract moral ideals that may be less cognitively accessible under strain. Drawing on this theoretical and empirical background, we designed a behavioral experiment to test the following hypotheses: (H1a) A crisis will induce higher support for efficiency-oriented principles in health care priority setting, and (H1b) there will be decreased support for equality-oriented principles compared with a noncrisis situation.

Policy shifts during the pandemic may not necessarily reflect changes in policymakers’ moral values but rather the limitations of preexisting ethical guidelines—particularly those expressed in abstract, general terms. Abstract formulations typically encapsulate core moral commitments—such as fairness, dignity, or need—while leaving room for contextual interpretation.^32^ These are often well-suited to stable and deliberative environments. However, in crises, where decisions must be made rapidly and under uncertainty, abstract principles may seem too vague to guide concrete action. In such contexts, more specific and action-oriented guidelines are likely to gain prominence. This distinction is supported by research in behavioral ethics and construal level theory, which shows that abstract language promotes equality-oriented, value-based thinking, whereas concrete language fosters instrumental reasoning and prioritization of efficiency.^5,20–22,31^ Thus, the emphasis on efficiency in many pandemic-era policies may not have stemmed solely from the urgency imposed by scarcity but also from the use of more concrete formulations of ethical principles—formulations that tend to foreground feasibility and cost-effectiveness and thereby make efficiency-based judgments more cognitively accessible and socially acceptable in crisis settings.

The Swedish Ethical Platform provides a particularly illustrative case for examining how abstraction and context influence support for ethical principles, as it explicitly includes both efficiency- and equality-oriented principles within a unified normative framework. Sweden’s initiative toward open and systematic health care rationing began during the economic downturn of the early 1990s, when a national consensus emerged around the necessity of rationing care. To ensure legitimacy and transparency, a parliamentary priorities commission was tasked with identifying ethical principles that could guide decision making in health care. Its work culminated in the establishment of a formal ethical platform, codified in Government Bill 1996/97:60,^33^ which has since served as the normative foundation for Swedish health care prioritization.

The ethical platform consists of three core principles: 1) the *human dignity principle*, which affirms that all individuals have equal value and rights regardless of personal characteristics or social status; 2) the *needs-solidarity principle*, which holds that resources should be directed toward those with the greatest needs; and 3) the *cost-effectiveness principle*, which stipulates that resources should be used efficiently, so long as this does not undermine basic health-related duties. Consistent with common distinctions in the literature, the human dignity and needs-solidarity principles are generally considered equality oriented: the former emphasizes equal moral worth, and the latter gives priority to the worse off. In contrast, the cost-effectiveness principle is efficiency oriented, as it focuses on optimizing resource use to maximize health outcomes. These principles are hierarchically ordered: the human dignity principle should be considered before the needs-solidarity principle, which in turn takes precedence over cost-effectiveness. Moreover, each principle was presented in both an abstract version—reflecting the official formulations in the Swedish ethical platform—and a more concrete version, derived from explanatory sections in the legislative proposition underlying the platform.^33^ For example, the needs-solidarity principle was formulated abstractly as follows: *Resources should be distributed according to need*. In the concrete version, this was expressed as: *More resources should be given to those with the most severe illnesses and worst quality of life*. This duality allows us to test not only whether support for ethical principles changes in a crisis but also whether the form of their presentation—abstract versus concrete—modulates public support.

Building on this framework, we predicted that support for equality-oriented principles—the human dignity and needs-solidarity principles—would be stronger when presented in abstract terms (Hypothesis H2) as such formulations are more likely to invoke general moral commitments to fairness and justice. In contrast, we expected that the efficiency-oriented cost-effectiveness principle would receive greater support when presented in concrete, action-oriented terms that emphasize practical constraints and resource optimization (Hypothesis H3).

## Methods

### Participants and Procedure

Hypotheses and main analyses were preregistered before data collection. The preregistration along with data and analysis codes can be accessed via this repository https://osf.io/f76us/. The online experiment was programmed in Qualtrics, and the data were collected in March 2023. We recruited 1,404 English-speaking, UK-based participants through Prolific,^26^ an online platform that connects researchers with a diverse pool of prescreened participants, enabling high-quality data collection across various demographics.^34^ The sample was 59% female, with a mean age of 39.90 years (SD = 13.13). Participants received a £2 reimbursement.

### Experimental Design and Materials

We opted for a between-subjects behavioral survey experiment using Likert-type agreement scales to assess support for each principle independently. This approach allowed us to examine absolute levels of moral endorsement across conditions, without requiring participants to make explicit trade-offs between principles. While trade-off methods such as conjoint or ranking designs^35^ are often used for priority-setting research, they risk inducing prominence effects—in which participants disproportionately favor options aligned with the most salient value, regardless of their prior stated preferences.^36^ Such designs may thus generate comparative judgments that obscure subtle shifts in the support for individual principles, which was the primary focus of our study.

Participants were randomly assigned to 1 of 4 experimental conditions in a 2 × 2 factorial design, crossing *crisis* versus *noncrisis* context with *abstract* versus *concrete* formulation of ethical principles. The crisis manipulation was implemented through a short scenario preceding the ethical principles. In the crisis condition, participants read the following:

*Imagine the following scenario: Your country is suffering from a large-scale natural catastrophe. Due to this, it is no longer possible for the health care system to treat everyone in need of health care. To what extent do you agree with the following principles as a basis for health care priority setting?*

In the noncrisis condition, this contextual preamble was omitted, and participants were asked:

*To what extent do you agree with the following principles as a basis for health care priority setting?*

All participants were then asked to indicate their level of agreement with 3 ethical principles on a 7-point Likert scale ranging from 1 = *strongly disagree* to 7 = *strongly agree*.

The ethical principles were based on the legislated Swedish ethical platform for health care priority setting,^37^ which consists of the human dignity principle, needs-solidarity principle, and cost-effectiveness principle. The principles were described either by using the abstract main definition from the legislative proposition or by replacing keywords from the definition with the concrete action-oriented descriptions derived from explanatory sections in the same legislative proposition.^33^ To ensure fidelity in translation and framing, the research team—drawing on its extensive experience with health care priority setting in the Swedish context—worked with an external collaborator with applied experience working with these principles in practice, to identify and adapt concrete definitions that accurately reflected key elements of the abstract formulations.

Table 1 displays the exact wording used. In the abstract condition, each principle included its official title (e.g., “Human Dignity Principle”), while in the concrete condition, these titles were omitted to focus participants on the explanatory content. For instance, the abstract version of the human dignity principle referenced “personal characteristics and functions in society,” while the concrete version replaced this with explicit examples: “talents, social position, income, age, etc.” Similarly, abstract references to distributing resources “according to needs” were made more concrete as “more resources are given to those with the most severe illnesses and worst quality of life.” The cost-effectiveness principle differed in whether it referred generally to “a reasonable relation between costs and effects” or specifically to “the use of a limited resource and what it overall provides.” All participants viewed the 3 principles simultaneously and in the same order, constituting a joint evaluation rather than a separate evaluation.^38^ In joint evaluations, options are assessed side by side, which can highlight contrasts and influence how attributes are weighted in moral or policy judgments.

**Table 1:** Abstract and Concrete Formulations of Ethical Principles

	Abstract	Concrete
Human dignity	**Human dignity principle:** All humans have the same value and the same right to care irrespective of **personal characteristics and function in society.**	All humans have the same value and the same right to care, irrespective of **their talents, social position, income, age, etc.**
Needs-solidarity	**Needs-solidarity principle:** Resources should be distributed **according to needs.**	Resources should be distributed **so more of the health care resources are given to those with the most severe illnesses and worst quality of life.**
Cost-effectiveness	**Cost-effectiveness principle:** In choices between different areas of operations or measures should a reasonable relation between **costs and effects**, measured in improved health or increased quality of life, be sought.	In choices between different areas of operations or measures should a reasonable relation between **the use of a limited resource and what it overall provides**, measured in improved health or increased quality of life, be sought.

Differences between the abstract and the concrete condition are **bolded**.

After completing the experiment, the participants answered demographic questions about their age, gender, and educational level (categorized as secondary or less, further education, or higher education). An attention check was included to assess response quality. One-way analyses of variance (ANOVAs) and chi-square tests confirmed no statistically significant demographic differences across the 4 experimental groups (see Table S1 in the Supplementary Materials), indicating successful randomization.

### Analysis

We defined three dependent variables corresponding to agreement levels for each ethical principle: human dignity, needs solidarity, and cost-effectiveness.

To test hypotheses H1a and H1b (regarding the effect of crisis versus noncrisis settings) and hypotheses H2 and H3 (regarding abstract versus concrete framing), we conducted 2-way ANOVAs for each dependent variable. These analyses assessed the main effects of the experimental factors as well as interaction effects. To evaluate robustness, we repeated the analyses while controlling for age, gender, and education. As an additional check, we reran the models excluding participants who failed the attention check. All statistical analyses were conducted using *R*.^39^ Full details, including code and experimental materials, are available in the project’s OSF repository https://osf.io/f76us/.

## Results

**Figure 1:**
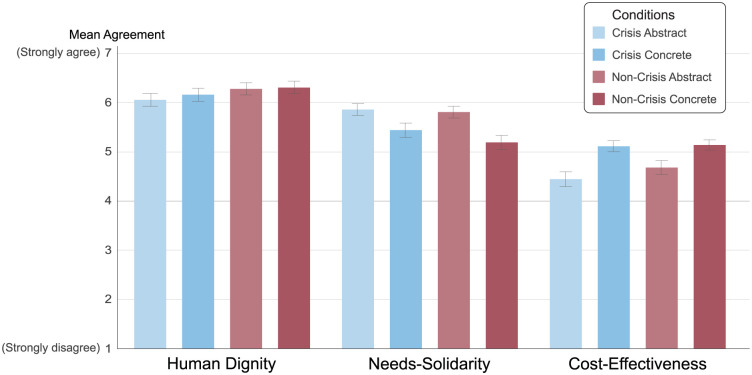
Mean agreement with each ethical principle in health care priority setting by experimental condition (*N* = 1,404). Bars indicate mean agreement ratings on a 7-point Likert scale (1 = *strongly disagree*, 7 = *strongly agree*) across the 4 experimental conditions (crisis v. noncrisis × abstract v. concrete). The 3 principles—human dignity, needs solidarity, and cost-effectiveness—were presented simultaneously. Error bars represent 95% confidence intervals.

Figure 1 presents the mean agreement levels across all experimental conditions. Across the full sample, the human dignity principle received the highest overall support as a basis for health care priority setting (M = 6.20, SD = 1.23), followed by the needs-solidarity principle (M = 5.58, SD = 1.28) and the cost-effectiveness principle (M = 4.84, SD = 1.28). These differences were statistically significant: support for the human dignity principle was higher than for the needs-solidarity principle, *t*(1,403) = 15.61, *p* < 0.001, and support for needs-solidarity was higher than for cost-effectiveness, *t*(1,403) = 16.39, *p* < 0.001.

### Crisis versus Noncrisis

Consistent with Hypothesis 1b, support for the human dignity principle was significantly lower in the crisis condition compared with the noncrisis condition (*F*[1, 1,401] = 8.56, *p* = 0.003, partial η^2^ = 0.006). This effect remained significant after including interaction terms (*F*[1, 1,400] = 8.56, *p* = 0.003) and demographic controls (*F*[1, 1,395] = 8.33, *p* = 0.004) but was attenuated when excluding participants who failed the attention check (*F*[1, 1,216] = 3.32, *p* = 0.069). Gender was also a significant predictor across models, with men (M = 5.97) expressing lower support than women (M = 6.35) (see Tables S2 to S5 in the Supplementary Materials).

In contrast to Hypothesis 1b, support for the needs-solidarity principle was significantly higher in the crisis condition (*F*[1, 1,401] = 5.15, *p* = 0.023, partial η^2^ = 0.004). This effect was robust across models including interaction terms (*F*[1, 1,400] = 5.15, *p* = 0.023), demographic controls (*F*[1, 1,395] = 4.83, *p* = 0.028) and when excluding participants who failed the attention check (*F*[1, 1,216] = 7.03, *p* = 0.008) (see Supplementary Tables S6 to S9 in the Supplementary Materials).

Contrary to Hypothesis 1a, support for the cost-effectiveness principle was lower in the crisis condition (*F*[1, 1,401] = 4.18, *p* = 0.041, partial η^2^ = 0.003). The effect persisted with interaction terms (*F*[1, 1,400] = 4.19, *p* = 0.041) but fell just short of conventional significance when controlling for demographics (*F*[1, 1,395] = 3.77, *p* = 0.052) and when excluding participants who failed the attention check (*F*[1, 1,216] = 3.42, *p* = 0.065). Across models, higher age was associated with greater support for cost-effectiveness (see Tables S10 to S13 in the Supplementary Materials).

### Abstract versus Concrete

There was no significant effect of abstractness on support for the human dignity principle (*F*[1, 1,401] = 1.04, *p* = 0.31, partial η^2^ = 0.001). This finding was similar across models with interaction terms (*F*[1, 1,401] = 1.01, *p* = 0.31), demographic controls (*F*[1, 1,395] = 1.55, *p* = 0.21), and when excluding participants who failed the attention check (*F*[1, 1,216] = 1.25, *p* = 0.26). Thus, Hypothesis 2 was not supported for the human dignity principle.

Support for the needs-solidarity principle was higher when presented in abstract terms (*F*[1, 1,401] = 60.68, *p* < 0.001, partial η^2^ = 0.042). This effect was robust across models with interaction terms (*F*[1, 1,400] = 61.10, *p* < 0.001), demographic controls (*F*[1, 1,395] = 61.40, *p* < 0.001), and when excluding participants who failed the attention check (*F*[1, 1,216] = 53.86, *p* < 0.001). Thus, Hypothesis 2 was supported for the need-solidarity principle.

Support for the cost-effectiveness principle was lower under abstract framing (*F*[1, 1,401] = 71.40, *p* < 0.001, partial η^2^ = 0.048). The effect was robust across models with interaction terms (*F*[1, 1,400] = 71.00, *p* < 0.001), demographic controls (*F*[1, 1,395] = 71.04, *p* < 0.001), and when excluding participants who failed the attention check (*F*[1, 1,216] = 65.49, *p* < 0.001). Thus, Hypothessis 3 was supported for the cost-effectiveness principle.

Full ANOVA tables, including interaction terms, covariates, and robustness checks, are available in the Supplementary Materials (Tables S2–S13).

## Discussion

In this study, we examined whether moral views about health care priority setting shift during a crisis and whether such shifts are driven by the situation itself or by the way in which ethical principles are framed. Using a preregistered experimental design and the 3 principles of the Swedish Ethical Platform—human dignity, needs solidarity, and cost-effectiveness—we found only limited support for what we have referred to as *crisis exceptionalism.* That is, while some differences emerged between crisis and noncrisis conditions, these were modest in size, and the overall ranking of principles remained stable. Instead, how ethical principles were formulated—abstractly or concretely—had a stronger and more consistent effect on public support.

We highlight three key findings for further discussion: (*i*) contrary to expectations, support for the needs-solidarity principle increased in a crisis; (*ii*) support for the cost-effectiveness principle decreased, rather than increased, in a crisis; and (*iii*) unlike the other two principles, support for the human dignity principle was unaffected by how it was framed.

### Reversals in Support for Needs and Cost-Effectiveness in a Crisis

Hypotheses H1a and H1b predicted that crisis settings would increase support for efficiency-oriented principles (cost-effectiveness) and reduce support for equality-promoting principles (human dignity and needs-solidarity). These expectations could not be confirmed (i.e., we were unable to reject the null hypothesis). Surprisingly, support for the needs-solidarity principle was higher in the crisis condition, while support for cost-effectiveness was lower.

This reversal raises interpretive challenges. One possibility is that participants interpreted “needs” in different ways. In the priority-setting literature, *needs* typically refer to either 1) *health care need*, defined as capacity to benefit, or 2) *health need*, defined as severity of illness or baseline disadvantage.^11,40,i^ While our abstract formulation allowed both interpretations, the concrete version emphasized severity and being worse off, which may have led participants to interpret *need* more in terms of health need. If so, the increased support for needs-solidarity during crisis may reflect a heightened concern for the worst off, even under resource constraints—a view that contrasts with some prior findings from the COVID-19 pandemic^13–17^ and behavioral experiments on scarcity.^26,27,30,31^

Similarly, the decline in support for cost-effectiveness during crisis contradicts expectations based on scarcity theory, which suggests that people under constraint are more likely to favor instrumental, outcome-maximizing policies. One possible explanation lies in the nature of these tasks. In many prior scarcity experiments, the objective is singular and efficiency oriented, such as minimizing total debt^22^ or maximizing the number of targets hit in an *Angry Birds*–style game.^27^ These scenarios offer clear, quantifiable goals and do not involve tradeoffs between competing moral values. In contrast, health care priority setting involves multiple, sometimes competing, objectives, such as needs-based allocation, equal access, and maximizing benefit, among which efficiency is only one. This moral pluralism makes the decision environment more ambiguous and normatively contested, which may in turn dilute the usual effects of scarcity.

Moreover, our “concrete” condition did not include explicit numerical outcomes (e.g., numbers of lives saved), as is common in many priority-setting studies using outcome-based trade-offs.^6,21,36,42^ This difference may help account for the lack of an expected increase in support for cost-effectiveness in the crisis condition: without clear outcome information, participants may have focused less on maximizing total health benefits and more on protecting the worst off. Put differently, when efficiency is presented as a general principle rather than through concrete numerical terms, its motivational power may be weaker.

### The Role of Abstractness in Shaping Support

In contrast to the limited and sometimes counterintuitive effects of the crisis context, the framing of ethical principles—as abstract or concrete—produced strong and consistent effects, confirming Hypotheses H2 and H3. Support for the needs-solidarity principle was significantly higher when presented in abstract terms, whereas support for the cost-effectiveness principle was higher when described concretely. These results align with prior research in behavioral ethics and construal level theory, which suggests that abstract language tends to activate broad moral commitments, such as fairness and equality, whereas concrete descriptions bring practical considerations and trade-offs into sharper focus.^5,21,22^

Beyond these framing effects, support for the human dignity principle was similar across levels of abstraction. One possible explanation is that this principle functions more like a formal justice norm—a moral rule that is more resistent to contextual reinterpretation. The abstract version emphasized universal rights and equal value, while the concrete version specified that care should not be based on characteristics such as talent, income, or age. The consistency in responses across framings suggests that participants endorsed both the high-level ideal and its specific implications. This stability may reflect the principle’s status as a broadly accepted moral baseline, less susceptible to pragmatic or contextual framing. Notably, this finding contrasts with earlier research showing that age is sometimes viewed as a relevant prioritization criterion,^43^ indicating that broad moral commitments may override such considerations when explicitly framed as matters of human dignity.

### Limitations

Several limitations should be noted. First, our crisis manipulation was based on a large-scale natural catastrophe, which may not generalize to other types of crises, such as pandemics, cyberattacks, or energy shortages. Different crises may trigger distinct psychological responses, especially when they vary in perceived causality, time horizon, or social proximity. That said, natural disasters are commonly used in public health and behavioral research because they evoke a clear sense of resource scarcity, urgency, and triage pressure without being politically charged or ideologically loaded. As such, they serve as a compelling test case for evaluating public attitudes toward prioritization in strained health systems.

Second, we used a UK-based sample to evaluate support for ethical principles formulated in Sweden. While this raises questions of contextual fit, the Swedish platform was chosen not to assess Swedish Ethical Platform per se but because it offers a well-defined, real-world framework with both abstract and concrete formulations, ideal for testing our hypotheses. The United Kingdom and Sweden share key institutional features, including universal, publicly funded health care systems that balance equity and efficiency. Nonetheless, differences in health policy discourse and crisis experience (e.g., during COVID-19) may influence how terms such as *needs* and *cost-effectiveness* are interpreted. We therefore caution against overgeneralization. Still, previous work with a Swedish sample^5^ found similar support patterns, suggesting some cross-contextual robustness.

Third, our use of joint evaluation—presenting all three principles simultaneously—may have influenced comparative judgments. While this mirrors real-world deliberations in ethics and policy, it may also amplify contrast effects or draw attention to specific trade-offs.

Fourth, although our abstract and concrete framings were constructed from authentic legislative texts and reviewed by experts, subtle wording differences may have influenced interpretation beyond abstraction per se. Finally, while our crisis and noncrisis scenarios were designed to simulate real constraints, their brevity and hypothetical nature limit psychological realism. More immersive or emotionally evocative crisis manipulations may yield different or stronger effects.

## Conclusion and Policy Implications

This study offers new insight into how crisis contexts and linguistic framing influence public support for core ethical principles in health care priority setting. Contrary to common assumptions about crisis-induced shifts toward efficiency, we found no consistent evidence that crisis alone reduces support for equality-oriented principles. Instead, our findings highlight the powerful role of formulation: abstract framings increased support for equality (particularly needs-solidarity), while concrete framings boosted support for efficiency (cost-effectiveness). These results suggest that how ethical guidelines are articulated may shape public receptivity as much as—if not more than—the situational context itself.

The findings have important implications for how ethical principles are communicated and operationalized in policy. Ethical frameworks are often developed in stable environments using abstract normative language. Yet, during crises, these principles must be translated into concrete, action-oriented policies. Our findings suggest that this translation process is not neutral: public endorsement of a principle can shift depending on how it is framed. Concretizing equity-oriented principles may lower their perceived legitimacy, whereas maintaining some level of abstraction may help preserve alignment with broader public values.

Thes results also carry broader relevance for policymakers, particularly in settings where legitimacy depends on public acceptance of priority-setting frameworks. In times of crisis, the need for transparent and publicly supported allocation principles becomes especially pressing. As governments and health authorities develop crisis preparedness strategies, these efforts may benefit from a clearer understanding of which principles the public finds justifiable, and how those principles should be communicated to foster public trust.

As health systems continue to face crises—from pandemics to climate-related disasters—understanding how people respond to both the content and presentation of ethical principles will be essential for crafting policy that is not only operationally effective but also morally and publicly sustainable.

## Supplemental Material

sj-docx-1-mdm-10.1177_0272989X251391177 – Supplemental material for Do Moral Views Change during a Crisis? An Experiment on Health Care Priority Setting Supplemental material, sj-docx-1-mdm-10.1177_0272989X251391177 for Do Moral Views Change during a Crisis? An Experiment on Health Care Priority Setting by Liam Strand, Erik Gustavsson and Gustav Tinghög in Medical Decision Making

## Notes

i. In actual health care priority setting, however, needs-based claims usually combine both aspects: health care need (how much a person can benefit from treatment) and health need (how badly off the person is in terms of illness severity or disadvantage).^41^
