# Supplementary material for: Do Moral Views Change during a Crisis? An Experiment on Health Care Priority Setting
Source: Med Decis Making. 2025 Dec 11;46(4):471–9. doi: 10.1177/0272989X251391177 (PMC13062458; doi:10.1177/0272989X251391177)
Supplement: sj-docx-1-mdm-10.1177_0272989X251391177 – Supplemental material for Do Moral Views Change during a Crisis? An Experiment on Health Care Priority Setting [file sj-docx-1-mdm-10.1177_0272989X251391177.docx]

Do Moral Views Change During a Crisis? An Experiment on Health Care Priority Setting: Supplementary Materials

# Additional Analysis

A one-way ANOVAs showed that there was no statistical significant difference between the four conditions in age (*F*(3,1400) = 0.97, *p* = 0.41) while χ2-tests showed that there was no statistical differences in gender (χ2(3, n = 1404)=1.78, *p* = 0.62), education (χ2(6, n=1404)=7.81, p = 0.25), and the number of successful attention checks (χ2(3, n = 1404) = 0.58, p = 0.90).

| Table S1: Descriptive statistics |  |  |  |  |
| --- | --- | --- | --- | --- |
| Context | Non-crisis | Non-crisis | Crisis | Crisis |
| Abstractness Level | Abstract | Concrete | Abstract | Concrete |
| Human Diginity Principe, mean (SD) | 6.28 (1.16) | 6.31 (1.21) | 6.05 (1.26) | 6.16 (1.27) |
| Needs-Solidarity Principle, mean (SD) | 5.81 (1.14) | 5.19 (1.33) | 5.86 (1.17) | 5.44 (1.34) |
| Cost-Effectiveness Principe, mean (SD) | 4.68 (1.39) | 5.14 (1.04) | 4.44 (1.45) | 5.11 (1.06) |
| Age, mean (SD) | 40.89 (13.78) | 39.89 (12.97) | 39.33 (13.02) | 39.52 (12.78) |
| Male, % (n) | 39.47% (135) | 44.25% (154) | 41.11% (148) | 40.68% (144) |
| At most Secondary Education, % (n) | 22.51 (77) | 17.24% (60) | 20.83% (75) | 15.82% (56) |
| Further Education, % (n) | 26.02% (89) | 25.29% (88) | 23.06% (83) | 25.99% (92) |
| Higher Education, % (n) | 51.46% (176) | 57.47% (200) | 56.11% (202) | 58.19% (206) |
| Successful attention check | 88.01% (301) | 87.50% (315) | 87.36% (304) | 86.16% (305) |
| n | 342 | 348 | 360 | 354 |

## Human dignity principle

Table S2 displays a Two-Way ANOVA (type II) for the human dignity principle while Table S3 describes a Two-Way ANOVA (type III) for the human dignity principle which includes an interaction effect. These are interpretated in the manuscript.

| Table S2 Two-Way ANOVA (type II) for Human Dignity Principle | | | | |
| --- | --- | --- | --- | --- |
|  | SS | df | F | p |
| Crisis | 12.87 | 1 | 8.56 | 0.003 |
| Concrete | 1.56 | 1 | 1.04 | 0.31 |
| Residuals | 2106.85 | 1401 |  |  |

| Table S3 Two-Way ANOVA (type III) for Human Dignity Principle | | | | |
| --- | --- | --- | --- | --- |
|  | SS | df | F | p |
| Crisis | 12.87 | 1 | 8.56 | 0.003 |
| Concrete | 1.52 | 1 | 1.01 | 0.31 |
| Crisis*Concrete | 0.81 | 1 | 0.54 | 0.46 |
| Residuals | 2106.00 | 1400 |  |  |

Table S4 displays a Two-Way ANOVA for the human dignity principle including the interaction effect and control variables. Notably, being male was statistically significant, where males were on average 5.97 points supportive of the principle while females where 6.35 points supportive of the principle. The interaction effect between crisis and abstractness level was not statistically significant.

| Table S4 Two-Way ANOVA for Human Dignity Principle | | | | |
| --- | --- | --- | --- | --- |
|  | SS | df | F | p |
| Crisis | 12.21 | 1 | 8.33 | 0.004 |
| Concrete | 2.27 | 1 | 1.55 | 0.21 |
| Age | 4.67 | 1 | 3.19 | 0.074 |
| Male | 42.94 | 1 | 29.29 | <0.001 |
| Education level | 7.87 | 2 | 2.69 | 0.069 |
| Crisis*Concrete | 0.38 | 1 | 0.26 | 0.61 |
| Residuals | 2046.2 | 1396 |  |  |

As seen in Table S5, the result remained similar when excluding participants who had failed the attention check.

| Table S5 Two-Way ANOVA for Human Dignity Principle Excluding Failed Attention Check | | | | |
| --- | --- | --- | --- | --- |
|  | SS | df | F | p |
| Crisis | 4.75 | 1 | 3.32 | 0.069 |
| Concrete | 1.79 | 1 | 1.25 | 0.26 |
| Age | 4.44 | 1 | 3.11 | 0.078 |
| Male | 48.13 | 1 | 33.66 | <0.001 |
| Education level | 7.58 | 2 | 2.65 | 0.071 |
| Crisis*Concrete | 2.11 | 1 | 1.48 | 0.22 |
| Residuals | 1740.5 | 1217 |  |  |

## Needs-solidarity principle

Table S6 displays a Two-Way ANOVA (type II) for the needs-solidarity principle while Table S7 describes a Two-Way ANOVA (type III) for the needs-solidarity principle which includes an interaction effect. These are interpretated in the manuscript.

| Table S6 Two-Way ANOVA (type II) for Needs-Solidarity Principle | | | | |
| --- | --- | --- | --- | --- |
|  | SS | df | F | p |
| Crisis | 8.01 | 1 | 5.15 | 0.023 |
| Concrete | 94.41 | 1 | 60.68 | <0.001 |
| Residuals | 2179.64 | 1401 |  |  |

| Table S7 Two-Way ANOVA (type III) for Needs-Solidarity Principle | | | | |
| --- | --- | --- | --- | --- |
|  | SS | df | F | p |
| Crisis | 8.01 | 1 | 5.15 | 0.023 |
| Concrete | 94.99 | 1 | 61.11 | <0.001 |
| Crisis*Concrete | 3.31 | 1 | 2.12 | 0.14 |
| Residuals | 2106.00 | 1400 |  |  |

Table S8 displays a Two-Way ANOVA for the needs-solidarity principle including the interaction effect and control variables. There were no statistically significant control variables. The interaction effect between crisis and abstractness level was not statistically significant.

| Table S8 Two-Way ANOVA for Needs-Solidarity | | | | |
| --- | --- | --- | --- | --- |
|  | SS | df | F | p |
| Crisis | 7.51 | 1 | 4.83 | 0.028 |
| Concrete | 95.39 | 1 | 61.40 | <0.001 |
| Age | 4.95 | 1 | 3.18 | 0.075 |
| Male | 0.91 | 1 | 0.59 | 0.44 |
| Education level | 0.96 | 2 | 0.31 | 0.74 |
| Crisis*Concrete | 3.67 | 1 | 2.36 | 0.12 |
| Residuals | 2168.8 | 1396 |  |  |

Two results changed when excluding participants who had failed the attention check (Table S9). First, the interaction effect between crisis and abstractness level was statistically significant (F(1,1216)=5.33, p=0.021). The means were the following: non-crisis abstract 5.81 (SD=1.15), non-crisis concrete 5.13 (SD=1.32), crisis abstract 5.85 (SD=1.18), crisis concrete 5.48 (SD=1.35). Thus, participants in the non-crisis condition were more affected by the abstractness level than participants in the crisis condition. Moreover, age was statistically significant. An ordinary linear regression with needs as outcome variable and age as control variable showed that a higher age was associated with a lower support for the needs-solidarity principle.

| Table S9 Two-Way ANOVA for Needs-Solidarity Principle Excluding Failed Attention Check | | | | |
| --- | --- | --- | --- | --- |
|  | SS | df | F | p |
| Crisis | 11.01 | 1 | 7.03 | 0.008 |
| Concrete | 84.33 | 1 | 53.86 | <0.001 |
| Age | 6.27 | 1 | 4.00 | 0.046 |
| Male | 0.50 | 1 | 0.32 | 0.57 |
| Education level | 0.31 | 2 | 0.10 | 0.91 |
| Crisis*Concrete | 7.79 | 1 | 4.97 | 0.026 |
| Residuals | 1905.4 | 1217 |  |  |

## Cost-effectiveness

Table S10 displays a Two-Way ANOVA (type II) for the cost-effectiveness principle while Table S11 describes a Two-Way ANOVA (type III) for the cost-effectiveness principle which includes an interaction effect. These are interpretated in the manuscript.

| Table S10 Two-Way ANOVA (type II) for Cost-Effectiveness Principle | | | | |
| --- | --- | --- | --- | --- |
|  | SS | df | F | p |
| Crisis | 6.54 | 1 | 4.18 | 0.041 |
| Concrete | 111.79 | 1 | 71.40 | <0.001 |
| Residuals | 2193.41 | 1401 |  |  |

| Table S11 Two-Way ANOVA (type III) for Cost-Effectiveness Principle | | | | |
| --- | --- | --- | --- | --- |
|  | SS | df | F | p |
| Crisis | 6.55 | 1 | 4.19 | 0.041 |
| Concrete | 111.04 | 1 | 71.00 | <0.001 |
| Crisis*Concrete | 3.94 | 1 | 2.52 | 0.11 |
| Residuals | 2189.00 | 1400 |  |  |

Table S12 displays a Two-Way ANOVA for the cost-effectiveness principle including the interaction effect and control variables. Age was statistically significant, and an ordinary linear regression showed that a higher age was associated with a higher support for the cost-effectiveness principle (beta = 0.010, SE=0.003, p<0.001). The interaction effect between crisis and abstractness level was not statistically significant.

| Table S12 Two-Way ANOVA for Cost-Effectiveness Principle | | | | |
| --- | --- | --- | --- | --- |
|  | SS | df | F | p |
| Crisis | 5.83 | 1 | 3.77 | 0.052 |
| Concrete | 109.74 | 1 | 71.04 | <0.001 |
| Age | 28.29 | 1 | 18.32 | <0.001 |
| Male | 1.25 | 1 | 0.81 | 0.37 |
| Education level | 6.15 | 2 | 1.99 | 0.14 |
| Crisis*Concrete | 3.76 | 1 | 2.44 | 0.12 |
| Residuals | 2156.4 | 1396 |  |  |

As seen in Table S13, the results remained robust when excluding participants who had failed the attention check.

| Table S13 Two-Way ANOVA for Cost-Effectiveness Principle Excluding Failed Attention Check | | | | |
| --- | --- | --- | --- | --- |
|  | SS | df | F | p |
| Crisis | 5.26 | 1 | 3.42 | 0.065 |
| Concrete | 100.97 | 1 | 65.49 | <0.001 |
| Age | 25.24 | 1 | 16.37 | <0.001 |
| Male | 3.2 | 1 | 2.07 | 0.15 |
| Education level | 7.64 | 2 | 2.48 | 0.084 |
| Crisis*Concrete | 5.04 | 1 | 3.27 | 0.071 |
| Residuals | 1876.4 | 1217 |  |  |

# Transcript of Survey

INSTRUCTIONS FOR ALL CONDITIONS

Welcome!

If you agree to participate in this study, we will ask you several questions related to your attitudes towards prioritisation of limited health care resources in different circumstances.

Your answers will be treated so no unauthorised persons access them. All publication of this research will only report results on an aggregated level or completely anonymized examples which will not identify you.

Participation in the study should take about 10 minutes and you will receive a participation fee of £2 for completing the entire study.

Please note: During the survey, there will be an attention check to ensure that you are paying attention to the study instructions.

There are no foreseeable risks to your participation in this study. By clicking the button below, you indicate that you understand the information that was presented and that your participation is voluntary, and you may withdraw your consent and discontinue participation in the project at any time.

- I consent to participate in this study

Please enter your Prolific ID

________________________________________________________________

[NEW SCREEN]

## [The non-crisis condition – high abstractness level]

For the next part of the survey, you will be asked about your preferences for different principles when prioritizing health care resources under certain circumstances.

Keep in mind that there are no right or wrong answers and please consider the information in the scenario carefully prior to giving your answers to each question.

[NEXT SCREEN]

To what extent do agree with the following principles as a basis for health care priority setting?

Human dignity principle: All humans have the same value and the same right to care independent of personal traits and function in society

- 1=Strongly disagree
- 2
- 3
- 4
- 5
- 6
- 7=Strongly agree

Needs-solidarity principle: Resources should be distributed according to needs.

- 1=Strongly disagree
- 2
- 3
- 4
- 5
- 6
- 7=Strongly agree

Cost-effectiveness principle: In choices between different areas of operations or measures should a reasonable relation between cost and effects, measured in improved health our increased quality of life, be sought.

- 1=Strongly disagree
- 2
- 3
- 4
- 5
- 6
- 7=Strongly agree

[NEW SCREEN]

## [The non-crisis condition – low abstractness level]

For the next part of the survey, you will be asked about your preferences for different principles when prioritizing health care resources under certain circumstances.

Keep in mind that there are no right or wrong answers and please consider the information in the scenario carefully prior to giving your answers to each question.

[NEXT SCREEN]

To what extent do agree with the following principles as a basis for health care priority setting?

All humans have the same value and the same right to care independent of their talents, social position, income, age etc.

- 1=Strongly disagree
- 2
- 3
- 4
- 5
- 6
- 7=Strongly agree

Resources should be distributed so more of the healthcare resources are given to those with the most severe illness and worst quality of life.

- 1=Strongly disagree
- 2
- 3
- 4
- 5
- 6
- 7=Strongly agree

In choices between different areas of operations or measures should a reasonable relation between the use of a limited resource and what it overall provides, measured in improved health our increased quality of life, be sought.

- 1=Strongly disagree
- 2
- 3
- 4
- 5
- 6
- 7=Strongly agree

## [The crisis condition – high abstractness level]

For the next part of the survey, you will be asked about your preferences for different principles when prioritizing health care resources under certain circumstances.

Keep in mind that there are no right or wrong answers and please consider the information in the scenario carefully prior to giving your answers to each question.

[NEXT SCREEN]

Imagine the following scenario: Your country is suffering from a large-scale natural catastrophe. Due to this, it is no longer possible for the health care system to treat everyone in need of health care. To what extent do you agree with the following principles as a basis for health care priority setting?

Human dignity principle: All humans have the same value and the same right to care independent of personal traits and function in society

- 1=Strongly disagree
- 2
- 3
- 4
- 5
- 6
- 7=Strongly agree

Needs-solidarity principle: Resources should be distributed according to needs.

- 1=Strongly disagree
- 2
- 3
- 4
- 5
- 6
- 7=Strongly agree

Cost-effectiveness principle: In choices between different areas of operations or measures should a reasonable relation between cost and effects, measured in improved health our increased quality of life, be sought.

- 1=Strongly disagree
- 2
- 3
- 4
- 5
- 6
- 7=Strongly agree

## [The crisis condition – low abstractness level]

For the next part of the survey, you will be asked about your preferences for different principles when prioritizing health care resources under certain circumstances.

Keep in mind that there are no right or wrong answers and please consider the information in the scenario carefully prior to giving your answers to each question.

[NEXT SCREEN]

Imagine the following scenario: Your country is suffering from a large-scale natural catastrophe. Due to this, it is no longer possible for the health care system to treat everyone in need of health care. To what extent do you agree with the following principles as a basis for health care priority setting?

All humans have the same value and the same right to care independent of their talents, social position, income, age etc.

- 1=Strongly disagree
- 2
- 3
- 4
- 5
- 6
- 7=Strongly agree

Resources should be distributed so more of the healthcare resources are given to those with the most severe illness and worst quality of life.

- 1=Strongly disagree
- 2
- 3
- 4
- 5
- 6
- 7=Strongly agree

In choices between different areas of operations or measures should a reasonable relation between the use of a limited resource and what it overall provides, measured in improved health our increased quality of life, be sought.

- 1=Strongly disagree
- 2
- 3
- 4
- 5
- 6
- 7=Strongly agree

## Experimental instructions (demographics)

INSTRUCTIONS FOR ALL CONDITIONS

[NEW SCREEN]

How old are you?

(The participants dragged a slider ranging from 18-100 with intervals of to indicate their age)

What’s your gender?

- Male
- Female

What’s your highest completed education?

- Primary education
- Secondary education (completed year 13)
- Further education (post-secondary and tertiary education)
- Higher education (at least Bachelor’s degree)

[NEW SCREEN]

We thank you for your time spent taking this survey.

Your response has been recorded.
